# Malarial Fever in Horses

**Published:** 1890-12

**Authors:** Gerald E. Griffin

**Affiliations:** Veterinarian, Fifth United States Cavalry


### MALARIAL FEVER IN HORSES.

By Gerald E. Griffin, D.V.S.,
*Veterinarian, Fifth United States Cavalry.*

During the past two summers my attention was forcibly attracted by a peculiar disease or condition of quite a number of horses which, though comparatively puzzling at first, was, after observation, diagnosed as a type of malaria. That this diagnosis is correct there is no doubt, and when the facts in the case have been carefully studied the evidence will bear out the conclusion.

The past summer here—Indian Territory—was a hot, dry and long one, temperature frequently reaching 104° F., and seldom falling below 85° except during the night. The station, Fort Reno, is situated on a slight eminence, surrounded on the north and east by low-lying flat lands through which flow the waters of the north fork of the Canadian River; the station drainage is good, at least that portion of it not connected with the stables, but the drainage from the latter is surface and very unsatisfactory. During the late spring, rains were not frequent but heavy, and the water collecting on the flats, surrounding the station to a depth of from six to eight inches, remained there until evaporated or absorbed. As the summer advanced the water supply of the garrison, whose source is the Canadian, became less abundant and less pure. The comparative impurity being due to the continual bathing in the river, above the source of garrison supply, of hundreds of Indians who congregate along its branch during the summer months, accompanied by their herds of ponies and troops of dogs; eventually the flow of water ceased altogether, thus forcing the cavalry horses, Fifth Cavalry, to procure their drinking water in the holes or pools in which it had collected along the river bed. These pools on being agitated gave off a very disagreeable odor, and in fact the majority of the animals refused to partake of the water for at least twenty-four hours. The main sewer of garrison empties itself into the above-mentioned stream about one mile below the post. This sewer emitted a vile odor from the manholes along its course, and particularly at its outlet, and as the fluids therefrom percolated through the exposed land of the river bed, the stench could be observed for upward of a mile down the course of the stream; fortunately, however, the prevailing winds were southerly, so that the effects of the effluvia were not felt to their fullest extent in the garrison. About this time malaria in its most severe and malignant form began to make its appearance in the abodes of the members of the post and in the immediate vicinity, four cases dying, while an alarming number were on the verge of collapse. It was at this stage the horses began to show the symptoms described.

Veterinary literature being almost silent on the subject of malaria, I was forced to look up data elsewhere, and am indebted to Hertz and Ziemssen for much valuable information in this connection. "The history of this disease reaches back to the earliest period of medical science" (Hertz), and the distinction between the various types quotidian, tertian and quartan, etc., were known and studied to a certain extent, but it was not until the introduction of cinchona bark from Peru and Spain, 1640, that any con-iderable degree of attention was given to the study of it. This study was now stimulated by the conflicting results following the use of the new drug, but no reliable results were reached in practice until after a druggist's clerk, Tabor, of Cambridge, had introduced the use of larger doses and more effective forms of administration; and until Sydenham conceived the idea of giving the cinchona immediately after the first attack, during the intermission, for the purpose of forestalling a subsequent paroxysm.

"Malarial affections comprise a class of diseases that are usually marked by a perceptible, after a very considerable, rise of temperature, and which are, furthermore, to be regarded as constituting different forms of one and the same morbid process because of their origin in the same purely *miasmatic* causes, the fact of their frequent occurring simultaneously within the same area, the nature of their anatomical products, as well as certain peculiarities characteristic of their course."

There is no part of the torrid or temperate zones in which greater or smaller areas of country may not be found where malaria prevails every year to a greater or less extent. It is well known in nearly all the countries of Europe, especially along the shores of the Baltic North German flatlands, the marshy plains of the Rhine and Holland. In England the disease is seldom encountered, and then on the eastern coast and on some of the flats of the Thames. Scotland and Ireland are free from it. It is claimed that Italy is, beyond question, the most malarious of all European countries. It is well known in Africa, notably along the river regions. In Asia a very malarious region is to be found in the river districts which are annually overflowed by the water of the streams. In Australia it is also found, though in a mild form. In the United States malarial affections present themselves often in very malignant form in the southern states, and are found throughout the country except in the extreme north.

Malarial diseases are rarely sporadic in character, and rarely advance over a large region of a country in the form of an epidemic or epizootic, but confine themselves to a certain district, thus giving them an endemic or enzootic character. Their endemic occurrence is especially common in marshy regions, but all marshes do not bear this relation to disease, and there are extensive swampy regions, even in hot climates, that are entirely free from malarial fever. Their influence varies with the amount of water they contain. Where the latter stands high, fevers are more rare; where the marshy ground is covered only by a thin sheet of water, which is exposed to the heating influence of the sun, malarial diseases are shown to abound, as the decomposition of organic matter, and especially of vegetable matter, seems to aid in their production. So that the most favorable conditions for the development of this poison are offered by marshes or swamps that have dried up, while their injurious influence is materially diminished as soon as heavy rains once more submerge the previously parched surface of the ground; yet instances occur in which, with every condition present for the development of malaria, this poison is entirely lacking. Some writers account for these exceptions on the ground of the disinfecting properties of ozone, which is claimed to be largely developed in some marshes, but ozone is very useful as a cloak for a great many theories.

The fact that marshes have a causative relation to malaria may be proved, according to some authorities, by the disappearance of the latter when marshes are drained dry and cultivated, and its reappearance when they are allowed to relapse into a state of nature.

The second important factor in the development of malaria is heat, for it is well known that the development of the disease in human practice usually takes place during the summer months, and that it disappears—or, at least, no new cases arise, during the winter, unless the latter is very mild. It is also an established fact that in malarious districts the disease increases materially during hot, dry months that have been preceded by moisture, and that it is greatly diminished as soon as fresh, strong currents of air, accompanied by rain, cleanse the atmosphere.

The fact that during the enzootic prevalence of malaria at this post only a certain number of animals were attacked by it—the majority remaining exempt, although subject to the same climatic influences—forces one to search for individual predisposing causes which may account for the difference. Previous attacks of fever constitute the greatest predisposing cause for renewed attacks of the same; whence we must conclude that after the first invasion some changes, unknown to us, are wrought in the organism, which are capable, without any immediate manifestations, of leading to a new outbreak of the disease on the slightest provocation, and which gradually disappear if no new infection takes place. Then, again, we have the long list of debilitating diseases as rendering the individual a congenial abode for the miasmatic poison. From a few observations which I made the following facts were adduced :

1st. That debilitated animals which were allowed to herd in the vicinity of the above-mentioned flats developed the disease in from twelve to fourteen days.

2d. Animals in robust health, with good appetites, which were allowed to herd and given plenty of exercise, resisted the disease.

3d. Animals in good health, but which were confined and allowed but little exercise, when permitted to run in the vicinity of flats, developed the disease in from ten to sixteen days.

4th. That there is a period of incubation, and that this period averages about thirteen days.

Attacks of simple, benign malarial fever observed by me made their appearance rather suddenly, with nearly the following train of symptoms in each case : The animal was reported "off his feed" at either the morning or evening meal. An examination was made, when it was found, in all cases except two, that the temperature had run up to 105° and 106° F ; pulse, 60 to 65 ; respiration, rather hurried, 15 to 20. Coat appeared a little dry, mouth hot and clammy, with a sour smell from breath (this indicating digestive troubles) ; head drooping, as if animal was affected with headache, and the whole appearance sluggish and drowsy. This condition of affairs continued for from three to five days, when the temperature gradually diminished until it reached the normal, the patient regaining his appetite for a few days, when the temperature would suddenly rise again to 106°, and the same appearances as before would be repeated.

In the two cases referred to above, the temperature of these animals was taken experimentally, with a number of others, at night, with negative results, and the following morning the temperature of these two cases had risen to 105° and 105.5° F., respectively, the animals showing the symptoms mentioned above. It should be remarked that a thorough examination of respiratory and digestive apparatus had been made in each and every case. The feces were natural, but perhaps a little foul smelling ; urine, when it could be observed, healthy, and, on being tested, appeared normal. Food consisted of the usual allowance of ten pounds of oats and twelve pounds of hay per day, half of oats being fed in morning and half in evening, while during the day the animals were herded. Water hard and fairly good. Salt allowance limited to twelve ounces per month per animal (which is entirely too small).

As the variety of drugs furnished by the army for veterinary use is small, I was at a loss how to treat the symptoms when they first appeared, and before an emphatic diagnosis was made. Aconite was tried, but it only tended to weaken the heart's action, without any other results. Stimulants, in the shape of alcohol, carbonate of ammonia and spiritus ether. nitros. were next made use of, with the effect that the appetite was stimulated, the patients partaking of soft bran mashes and a little hay, but the temperature remained the same. After a positive diagnosis had been made, quinine and antipyrine were purchased from individual funds, and administered in five cases with good results; but preference was given the quinine, as its action, while not being so pronounced as the antipyrine, still had a more permanent effect, and when given in large doses, ʒiij to ʒvj, immediately after the first rise of temperature subsided, prevented the recurrence of a subsequent paroxysm This the antipyrine failed to accomplish, although it reduced the then existing temperature more promptly, but at the same time seemed to weaken the action of the heart. Some of the cases which received no treatment other than with the drugs furnished by the government, continued on with attacks of fever, which appeared periodically for several months, pulling the animals down in flesh and appearance to an astonishing extent. The disease gradually disappeared in these cases, the intervals between the paroxysms becoming longer—three to five, to ten, to thirteen, to twenty days—until it failed to reappear entirely, the patients regaining in a surprising manner their lost condition and vigor.

If everything is taken into consideration—the dry season, the poor water and its scarcity for a short period, the defective stable drainage, the intense and continued heat, the poorly-flushed main sewer, with its stench, the low-lying flat lands, which held the water for a long time, and the decaying vegetable matter on those flats, and, above all and most important, the endemic character of pernicious malaria among the human family in the vicinity, together with the apparent disappearance of the disease on the approach of cooler weather and copious rains—I am satisfied that there can be but little doubt but the cases described were those of genuine malaria of the simple or benign form.

